# The Population Comparison Index: An Intuitive Measure to Calibrate the Extent of Impairments in Patient Cohorts in Relation to Healthy and Diseased Populations

**DOI:** 10.3390/ijerph20032168

**Published:** 2023-01-25

**Authors:** Götz Gelbrich, Caroline Morbach, Timo Deutschbein, Martin Fassnacht, Stefan Störk, Peter U. Heuschmann

**Affiliations:** 1 Institute of Clinical Epidemiology and Biometry, University Würzburg, 97080 Würzburg, Germany; 2 Comprehensive Heart Failure Center, University Hospital Würzburg, 97078 Würzburg, Germany; 3 Clinical Trial Center, University Hospital Würzburg, 97080 Würzburg, Germany; 4 Department of Medicine I, Division of Cardiology, University Hospital Würzburg, 97080 Würzburg, Germany; 5 Department of Medicine I, Division of Endocrinology and Diabetes, University Hospital Würzburg, 97080 Würzburg, Germany; 6 Medicover Oldenburg MVZ, 26122 Oldenburg, Germany

**Keywords:** reference data, normal values, disease severity, disease score, comparability

## Abstract

We assume that a specific health constraint, e.g., a certain aspect of bodily function or quality of life that is measured by a variable X, is absent (or irrelevant) in a healthy reference population (Ref0), and it is materially present and precisely measured in a diseased reference population (Ref1). We further assume that some amount of this constraint of interest is suspected to be present in a population under study (SP). In order to quantify this issue, we propose the introduction of an intuitive measure, the population comparison index (PCI), that relates the mean value of X in population SP to the mean values of X in populations Ref0 and Ref1. This measure is defined as PCI[X] = (mean[X|SP] − mean[X|Ref0])/(mean[X|Ref1] − mean[X|Ref0]) × 100[%], where mean[X|.] is the average value of X in the respective group of individuals. For interpretation, PCI[X] ≈ 0 indicates that the values of X in the population SP are similar to those in population Ref0, and hence, the impairment measured by X is not materially present in the individuals in population SP. On the other hand, PCI[X] ≈ 100 means that the individuals in SP exhibit values of X comparable to those occurring in Ref1, i.e., the constraint of interest is equally present in populations SP and Ref1. A value of 0 < PCI[X] < 100 indicates that a certain percentage of the constraint is present in SP, and it is more than in Ref0 but less than in Ref1. A value of PCI[X] > 100 means that population SP is even more affected by the constraint than population Ref1.

## 1. Introduction

The characterization of patients with a certain disease (condition A) may require quantifying specific descriptors of outcomes such as physical alteration, loss of function, or severity of limitations in daily living, and relating these patients to individuals who do not have condition A. The comparator could be a population of healthy individuals, allowing for an estimation of the extent of such a constraint in relation to what is considered the “normal” healthy status. Alternatively, the comparator could also be a population of individuals with a disease or a comorbidity of high prevalence (condition B, e.g., diabetes mellitus). This would allow for demonstrating how patients with condition A compare to a population with condition B with respect to a set of variables.

Now, we can consider a combination of both situations. We can assume that there is a population of healthy individuals and a population of individuals with condition B. Both populations have been characterized in a manner that facilitates direct comparisons. In particular, it is known that the mean value of a specific variable X (or the mean values of a set of variables) is significantly different in each population, and the magnitude of this difference is clinically or biologically meaningful. In the following, both populations, although representing a diametrically opposed health status, serve as reference populations. We can now aim to estimate in patients with condition A—who are known to frequently also suffer from condition B (or some constraint comparable to condition B)—how close or far apart they are from both healthy individuals and from patients with condition B, with respect to the variable(s) X.

Here, we introduce the concept of a new measure—which we termed population comparison index (PCI)—that allows quantifying and intuitively visualizing the “location” of patients with condition A relative to both healthy subjects and patients suffering from condition B.

For example, with respect to the quantitative variable(s) X, patients with condition A may be located:

(i) close to the healthy reference group but well apart from patients with condition B,
(ii) between the healthy and the diseased reference groups, i.e., close to either group or exactly halfway,
(iii) close to the diseased reference group but considerably far from the healthy reference group, or
(iv) beyond the diseased reference group (as judged from the viewpoint of the healthy reference group).

## 2. Definition and Interpretation

We can suppose Ref0 and Ref1 are well-characterized healthy and diseased reference populations, respectively, with different population means of a quantitative variable X. As well, X may be an indicator for a property (value of 0 = not present and value of 1 = present). Furthermore, we can suppose that SP is a study population of interest. Then, the PCI can be defined as:

PCI_X_ = (E[X|SP] − E[X|Ref0])/(E[X|Ref1] − E[X|Ref0]) × 100[%],

where E[X|.] is the expected value of the variable X in the respective population.

If it is known that the values of X depend upon certain covariables, for example, sex and age, one may wish to take this into account and compute a stratified version of the PCI. We can suppose that s(.) is a stratification function that assigns to each subject ω of the entire population a stratum s(ω) defined by the values of the covariables, for example, sex and age group. The stratified PCI is then defined as:

PCI_X,s_ = E_ω∈SP_[(X(ω) − E[X|Ref0∩s(ω)])/(E[X|Ref1∩s(ω)] − E[X|Ref0∩s(ω)])] × 100[%],

where E_ω∈SP_[.] is the expected value running over all patients ω in the study population SP. We note that the stratified version of the PCI only makes sense if E[X|Ref1∩s(ω)] − E[X|Ref0∩s(ω)] has the same sign and is significantly different from zero at a clinically meaningful extent in all strata s(ω), ω∈SP.

The interpretation is that PCI_X_ transforms E[X|SP] linearly to a scale with E[X|Ref0] as the zero and E[X|Ref1] as the unit. PCI_X_ ≈ 0 implies that the patient cohort of interest is similar to the healthy reference population, and there is no trend towards the diseased reference group with respect to X. PCI_X_ ≈ 100 means that the impairment relative to the healthy reference group measured by X is of a comparable magnitude in both the patients of interest and the diseased reference group. When 0 < PCI_X_ < 100, the patients of interest exhibit some impairment in X that is worse than in the healthy reference group but not as severe as in the diseased reference group. If PCI_X_ < 50, the patients of interest are more similar to the healthy reference group, and if PCI_X_ > 50, they are more similar to the diseased reference group. PCI_X_ ≈ 50 means that they are halfway in between. If X measures some impairment associated with the disease in the diseased reference group, then compared to healthy references, PCI_X_ is simply the percentage of that impairment, which is present in the cohort of interest. If PCI_X_ > 100, then the impairment experienced by the patients of interest is larger than that of the diseased reference group.

We note that a positive value of PCI_X_ always indicates that the population SP departs from normality as represented by Ref0 into the same direction, similar to Ref1. If increased values of X are associated with the disease defining Ref1, then E[X|Ref1] > E[X|Ref0], and PCI_X_ > 0 is equivalent to E[X|SP] > E[X|Ref0]. On the other hand, if the disease is associated with decreased values of X, then E[X|Ref1] < E[X|Ref0], and PCI_X_ > 0 holds if E[X|SP] < E[X|Ref0].

If PCI_X_ < 0, then one of the populations SP and Ref1 has increased values of X in comparison with healthy references Ref0 and the other has decreased values. This does not, however, mean that the cohort of interest SP is “more healthy” than Ref0 because deviation from normality into the opposite direction may be pathological, though with different underlying biological mechanisms and clinical consequences than in population Ref1.

## 3. Variants

Many variables in medicine and biology represent their information on a multiplicative (rather than an additive) scale. This means that it is not the same difference of arithmetic means, but rather, the same ratio of geometric means, which indicates the same difference in biological activity. Typical examples are the variables involved in signalling cascades and feedback loops, e.g., hormones, mediators of immune responses such as immune globulin concentrations, electroneurographic data such the period of latency, and many others. Such variables should be log-transformed, and for log(X), the same numerical difference, again, has the same biological meaning.

Statistics may help to discriminate the additive and the multiplicative nature of variables. If a variable is additive, i.e., if it is the sum of many small contributing values, its distribution is approximately normal (at least, in healthy populations). This is an immediate consequence of the central limit theorem. If the values of a variable X result from the multiplicative accumulation of many small contributions, then log(X) is the respective sum of many small contributing values, and hence, it is also approximately normally distributed. Therefore, the examination of whether X or log(X) is normally distributed may provide guidance when selecting the scale for expression of the information contained in X. If the nature of X is multiplicative, we should consider PCI_log(X)_ rather than PCI_X_. Both indices can always be numerically computed, and they may materially differ from each other, but the latter is likely biologically nonsensical when X exists in the multiplicative world.

An additional variant of PCI may be that instead of using the average of a diseased population E[X|Ref1] to define the unit of the scale for PCI, a diagnostic threshold T_X_(Ref0,Ref1) may be used. Such thresholds may be established in the diagnostic guidelines of specialist medical societies. They are based on comprehensive studies of non-diseased populations (Ref0) and diseased populations (Ref1). The recommendation for an individual ω is that if the individual value is below the threshold, i.e., if X(ω) < T_X_(Ref0,Ref1), then this supports the notion that the disease is not present; conversely, if X(ω) > T_X_(Ref0,Ref1), then this supports the notion that the disease is present and further diagnostic measures should be considered.

We can, therefore, define the threshold-based variant of PCI as:

PCI_T,X_ = (E[X|SP] − E[X|Ref0])/(T_X_(Ref0,Ref1) − E[X|Ref0]) × 100[%].

We note that E[X|Ref0] < T_X_(Ref0,Ref1) < E[X|Ref1], i.e., the diagnostic threshold is in between Ref0 and Ref1, and hence, PCI_T,X_ > PCI_X_.

## 4. Computation

We can assume that Ref0’⊂Ref0, Ref1’⊂Ref1, and SP’⊂SP are samples from both the reference populations and the patient population under investigation with sizes of N_0_, N_1_, and N_●_, respectively. We can let Ω = Ω_1_∪Ω_2_∪…∪Ω_q_ represent a partition of the entire population categorized into disjoint subsets called strata, and s:Ω→{1, …, q} represents the stratification function where s(ω) = k if ω∈Ω_k_ for all ω∈Ω. We can then denote by Ref0’_k_ = Ref0’∩Ω_k_ and Ref1’_k_ = Ref1’∩Ω_k_ the strata within the reference samples. We can let X represent a quantitative variable or an indicator variable of a property, and then we can suppose that the difference between the mean values mean[X|Ref0’] and mean[X|Ref1’] in the reference samples has satisfactory statistical significance and clinical (or biological) relevance.

For each subject in the study sample ω∈SP’, we can compute the individual PCI value as:

PCI_X_^(ω) = (X(ω) − Mean[X|Ref0’_s(ω)_])/(Mean[X|Ref1’_s(ω)_] − Mean[X|Ref0’_s(ω)_]) × 100[%].

Thus, we define a linear transformation of the real line where the average of the X values of the healthy reference subjects belonging to the same stratum as ω is mapped to zero and the average for the diseased reference subjects of that stratum is mapped to 1. PCI_X_^(ω) then represents this transformation applied to X(ω). For a quantitative variable X, PCI_X_^(ω) indicates how ω is placed between the healthy and the diseased reference subjects with respect to X.

The estimate of the population comparison index is computed as the average of the individual PCI values of all subjects in the study sample as follows:

PCI_X_^ = Mean[PCI_X_^(ω)|ω∈SP’].

The unstratified version is the special case with q = 1, Ω = Ω_1_, and a constant function s(ω) = 1.

If the reference sample sizes N_0_ and N_1_ are “very large”, mean[X|Ref0’] and mean[X|Ref1’] can be considered to be precise estimates of E[X|Ref0] and E[X|Ref1] with (practically) zero variance. Then, PCI_X_^ is computed from mean[X|SP’] by a linear transformation, and its confidence interval is obtained by applying the same transformation to the confidence limits for mean[X|SP’]. To determine what sample size can be considered “very large”, as a criterion, we suggest that E[X|Ref1] − E[X|Ref0] can be estimated up to a relative error of one percent at the 99 percent confidence level, or in other words, there is a probability of 99 percent that the distance between 0 and 100 on the scale for the PCI is determined with a precision of one percent. This yields the following condition:

515 × (Var^[X|Ref0’]/N_0_ + Var^[X|Ref1’]/N_1_)^0.5^ < Mean[X|Ref1’] − Mean[X|Ref0’],

where Var^[X|.] is the estimated variance of X in the respective sample. To provide a numeric idea of “very large” samples when assuming this condition, we can suppose that X is normally distributed with equal variances in both populations such that the 95th percentile of X in the healthy reference population corresponds to the fifth percentile in the diseased reference population and N_0_ ≈ N_1_. Then, samples of approximately 50,000 individuals in each population would fulfil the condition for being “very large”.

The threshold for the size of “very large” samples will depend upon the size of the overlap of the distribution of X in the healthy and the diseased reference populations and upon the desired precision of the definition of the unit on the PCI scale. We note that the latter is an arbitrary choice. If the reader prefers assumptions other than the one provided above, or if they want to avoid considering the estimates for E[X|Ref0] and E[X|Ref1] to be free of variance, they may proceed as described below, regardless of sample size considerations.

If N_0,_ N_1,_ or both are smaller than “very large”, or if the strata of a stratified version of PCI are no longer “very large”, or if the uncertainty of the estimates of E[X|Ref0] and E[X|Ref1] are to be taken into account regardless of sample sizes, we suggest using the bootstrap method [1] to compute the confidence interval for PCI_X_^, where bootstrapping is carried out on all three samples (Ref0’, Ref1’ and SP’). Technically, a bootstrap step is a random selection of the weight functions w_0_, w_1_, and w_●_ from Ref0’, Ref1’, and SP’, respectively, into the non-negative integers such that the sums of the weights in the three samples are equal to N_0_, N_1,_ and N_●_, respectively. For Δ = 0, 1, and ●, we can select a sampling function v_Δ_:{1, …, N_Δ_}→{1, …, N_Δ_}, where all possible sampling functions are equally likely to be chosen. For each k = 1 … N_Δ_, the weight w_Δ_(k) = #{m|v_Δ_(m) = k} is the number of occurrences of k in the image of v_Δ_. The bootstrap PCI_X,w_^ for the triple w = (w_0_, w_1_, and w_●_) is calculated from the respective weighted means using these weight functions. When a larger number of bootstrap steps (e.g., *n* = 1000) have been carried out, the lower 2.5 percent and the upper 2.5 percent of the PCI_X,w_^ values are removed and the span of the remaining 95 percent of these values is the 95 percent confidence interval for PCI_X_^.

Typically, the sizes of the strata in the investigated samples do not exactly match the sizes of the strata in the population. Even if they do match, e.g., because sampling occurred in a stratified fashion, the percentages of the strata in the population may vary over time. We, therefore, suggest not to fix the sizes of the strata for bootstrapping, i.e., not requiring that the sums of the weights w_0_, w_1_, and w_●_ in the strata should match the sizes of the strata in Ref0’, Ref1’, and SP’, respectively, but rather, that we let the sizes of the strata be subject to variability.

If a diagnostic threshold is being used instead of a diseased reference sample for Ref1’ (see section “Variants” above), then a constant is being used instead of the empirical mean[X|Ref1’], and hence, bootstrapping must be carried out only with the weight functions w_0_ and w_●_.

**Example 1**

Petersen et al. [2] reported a modest but significant decrease in cardiac, renal, and pulmonary function in 443 subjects after predominantly mild to moderate SARS-CoV2 infection (sample SP’) in comparison with 1328 individuals from a local population-based study (“healthy” sample Ref0’). The biomarker N-terminal pro B-natriuretic peptide (NT-proBNP) is secreted by cardiomyocytes in states of cardiac pressure and/or volume overload and, hence, serum levels are increased in individuals with cardiac and renal dysfunction. NT-proBNP was increased by a factor of 1.4 in the cohort of interest (88 ng/L in SP’ vs. 63 ng/L in Ref0’). This effect size was considered important since in routine heart failure treatment, intra-individual changes of up to 30 percent are regarded clinically relevant, see e.g., [3].

To estimate the clinical importance of the respective elevation of NT-proBNP in the cohort of interest, the relation to both healthy individuals and individuals with overt heart failure (e.g., the diseased reference sample) can be determined. For the diseased reference group Ref1’, we chose the study sample of the CIBIS-ELD trial [4] where *n* = 876. This trial recruited patients aged ≥65 years with chronic, yet symptomatic, stable heart failure. Per the selection criteria, these patients were older (≥65 years required) than those in the Ref0’ and SP’ populations. Because the time between diagnosis of heart failure and blood sampling was kept short in most cases, they could be compared to patients with newly diagnosed overt heart failure. In the CIBIS-ELD patients, the median NT-proBNP level was 609 ng/L (interquartile range of 255 to 1614 ng/L), i.e., it was lower than that observed in other trials investigating patients with stable chronic heart failure (where the typical median NT-proBNP levels were 2000 ng/L and above) [5,6].

The hormone brain-natriuretic peptide (BNP) is part of the regulation process of intravascular volume and blood pressure. BNP must be represented on a multiplicative scale as requires its prohormone, the amino-terminal fragment NT-proBNP, which is produced in a 1:1 ratio when pro-BNP is split into an active peptide (BNP) and an inactive remainder (NT-proBNP). Hence, we deal with the geometric mean of NT-proBNP in Ref1’, which can be estimated as the geometric mean of the median and the quartiles. Assuming an approximately log-normal distribution, this yields a value for NT-proBNP of 631 ng/L.

Due to the multiplicative nature of NT-proBNP, we can calculate PCI on the logarithmic scale as follows:

PCI_mult_^ = (log(88) − log(63))/(log(631) − log(63)) × 100 = 14.5

The interpretation is that the mean NT-proBNP level in SARS-CoV2 patients is located at a point covering 14.5% of the distance between a healthy population and patients with incipient heart failure. Put differently, these SARS-CoV2 patients completed approximately one-seventh of “the journey towards heart failure” (as measured by NT-proBNP).

Of course, we also can compute the additive version of PCI as follows:

PCI_add_^ = (88 − 63)/(631 − 63) × 100 = 4.4

However, as NT-proBNP behaves multiplicatively, this number is nothing but a certain amount of ink on paper and does not reflect the “true” biological distance between the patient populations.

As an alternative, we may instead use an accepted NT-proBNP threshold, e.g., in patients with suspected heart failure outside a hospital, the European Society of Cardiology recommends the application a threshold of ≤125 ng/L, indicating the absence of heart failure, whereas levels of >125 ng/L mandate further diagnostic work-up. This cut-off value has been validated and found to be useful [7]. The “threshold version” of PCI can then be computed (on the multiplicative scale) as follows:

PCI_threshold_^ = (log(88) − log(63))/(log(125) − log(63)) × 100 = 48.8.

This means that patients with former mild to moderate SARS-CoV2 infection have covered approximately “half of their journey” towards the NT-proBNP threshold where heart failure becomes more likely than unlikely. We note that the different values of PCI_mult_^ = 14.5 and PCI_threshold_^ = 48.8 are not contradictory, but they have different interpretations.

**Figure 1:**
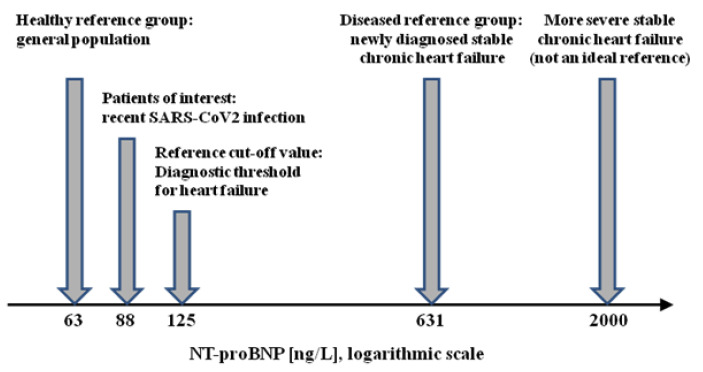
Schematic plot of the relevant NT-proBNP values for example 1.

A graphical illustration of the NT-proBNP values considered in this example is given in Figure 1.

**Example 2**

Cushing’s syndrome is caused by excess cortisol, inducing an increase in blood pressure, blood glucose, lipids, and body weight. These factors frequently lead to the development of a metabolic syndrome that is characterized by obesity, arterial hypertension, hyperlipidemia, and diabetes mellitus. Causal therapy and biochemical cures are thought to reduce the cardiovascular risk in these patients, although the long-term alterations in cardiac structure and function have not been studied. Here, we present data of 56 patients with cured endogenous Cushing’s syndrome (SP’) [8]. The reference samples consist of the participants of the population-based STAAB study [9,10] without metabolic syndrome (Ref0’; N_0_ = 4041) and those with metabolic syndrome (Ref1’; N_1_ = 924).

Glycosilated hemoglobin A1c (HbA1c) is a measure of hyperglycemia over the previous three months and, therefore, an indicator of the presence of diabetes mellitus and/or successful glycemic control. High values of HbA1c are unfavourable and will—in our example—be regarded as a surrogate marker of the metabolic syndrome.

Furthermore, the metabolic syndrome may result in cardiac remodelling and impaired ventricular function. Here, we show the data of left-ventricular posterior wall thicknesses (LVPW), which increase with chronic pressure overload, e.g., in arterial hypertension. An increased LVPW is considered an unfavourable sign as it indicates hypertrophy.

As a functional parameter, we chose E/e’, which was obtained from echocardiography. The numerator of this ratio represents the velocity of early diastolic left ventricular inflow, which depends on left atrial and left ventricular filling pressures (which is elevated in heart failure) and active left ventricular relaxation (which is reduced in heart disease). The denominator is a measure of left ventricular relaxation velocity (which is reduced in heart disease). Reduced relaxation velocity (e’) relative to a high left ventricular inflow velocity (E) indicates stiffness of the left ventricular myocardium, and hence, high values of E/e’ are considered to represent an impaired filling function of the left ventricle (which is unfavourable).

**Figure 2:**
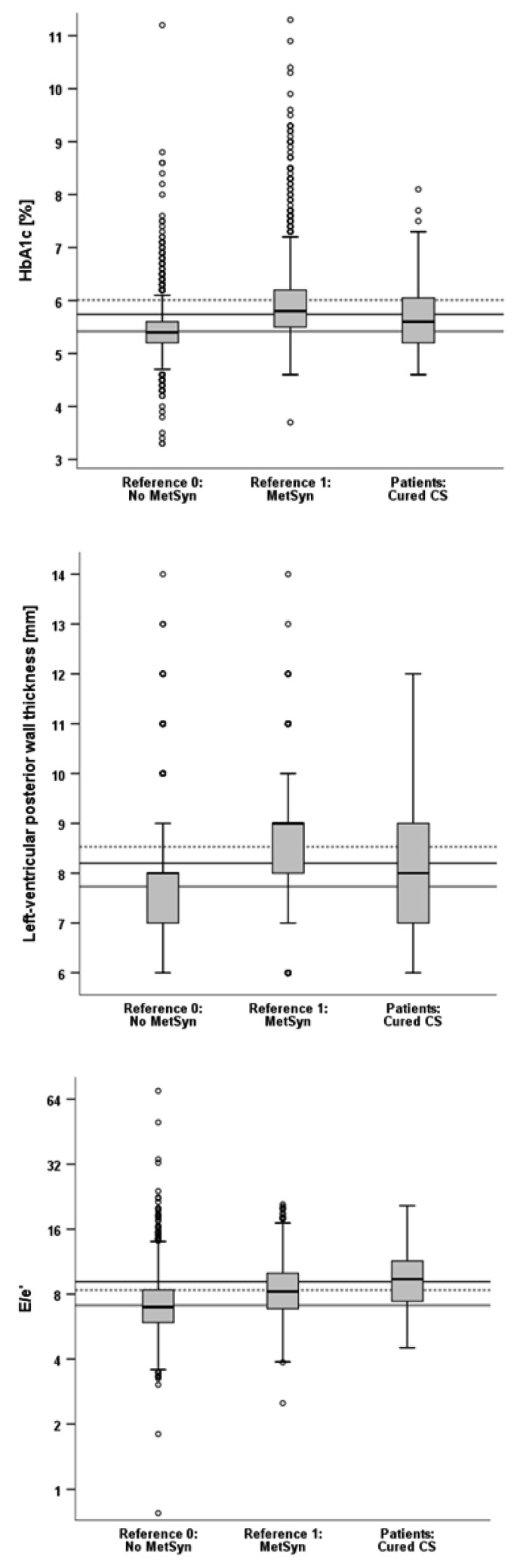
Boxplots of HbA1c, left ventricular posterior wall thickness, and the left ventricular inflow velocity to left ventricular relaxation velocity ratio E/e’ in the reference samples and in the patient sample of interest for example 2. The horizontal lines represent the overall non-stratified means (gray solid line, gray dotted line, and black line, respectively). MetSyn: metabolic syndrome, CS: Cushing’s syndrome.

The raw data used in this example for HbA1c, LVPW, and E/e’ are shown in Figure 2.

**Figure 3:**
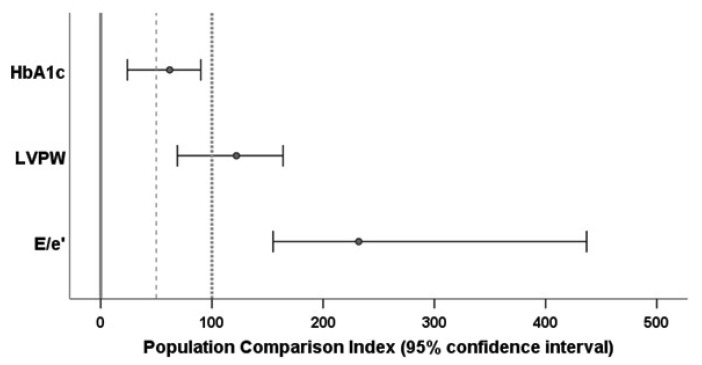
Stratified population comparison indices for HbA1c, left-ventricular posterior wall thickness (LVPW), and the left ventricular inflow velocity to left ventricular relaxation velocity ratio E/e’ and their 95 percent confidence intervals for example 2. The reference lines at 0 (solid) and 100 (dotted) indicate the means of the healthy and the diseased reference subjects, respectively. The threshold of 50 (dashed line) discriminates whether the patients of interest were closer to the healthy (<50) or the diseased (>50) references.

PCIs for all three variables were computed with stratification by age (≤55 and >55 years) and sex. As suggested by the distribution of the E/e’ data, the PCI for this variable was calculated on the logarithmic scale. The 95 percent confidence intervals for the PCI estimates were calculated by the bootstrap method with 1000 runs. The results are shown in Figure 3. Additional details can be found in the Supplementary Material.

As indicated by the confidence interval for PCI_HbA1c_^ being above 0, patients with cured endogenous Cushing’s syndrome retained a significant diabetic burden which, however, was lower than in the group with metabolic syndrome (with a confidence interval of below 100).

The confidence interval for PCI_LVPW_^ was above 50 and included the value 100, and so we would conclude that values of LVPW in patients with cured endogenous Cushing’s syndrome were comparable to those of the population with metabolic syndrome. Former Cushing patients were significantly more similar to the individuals with metabolic syndrome than to individuals without it. Figure 2 shows an overall mean LVPW of the Cushing sample between the means of the reference samples, which would lead to a PCI of between 0 and 100. However, this would be the value without stratification. When taking into account that 43 percent of the reference individuals were without metabolic syndrome but 69 percent of those with it were aged above 55 years, it is not surprising that the stratified estimate for the PCI became quite different from the non-stratified estimate.

The confidence interval for PCI_E/e’_^ was beyond 100. We thus would conclude that patients with endogenous Cushing’s syndrome (even after hypercortisolism had been cured) have a higher degree of left ventricular stiffness than the reference population with metabolic syndrome. Again, the data shown in Figure 2 suggest that the E/e’ values of the Cushing patients were closer to those of the references with metabolic syndrome than the E/e’ values of the healthy references were, which would lead to a PCI of between 100 and 200. However, this would be the non-stratified PCI; the stratified calculation yielded an index of above 200.

As this example shows, PCI is a technique that allows researchers to “compare apples with oranges” in some sense. Variables measured on completely different scales can be transformed to a scale with 0 for healthy reference groups and 100 for diseased reference groups. PCI quantifies how the percentage of the damage associated with a well-studied disease B is present in the patient cohort of interest with disease A. Different variables describe different aspects of the phenotype of disease A. The different PCI values of the particular variables allow researchers to judge which aspects of disease B are less or more present in the patients of interest with disease A. In our example, where metabolic syndrome was defined as disease B, the cured Cushing patients (disease A) were “halfway on their journey to disease B” with respect to HbA1c, similar to the diseased reference population with respect to ventricular hypertrophy and even worse with respect to ventricular stiffness.

## 5. Discussion

We propose PCI_X_ as a measure which simultaneously compares a patient population of interest to a healthy and to a diseased reference population with respect to a variable X. This measure is intuitive, and its estimate (jointly with its confidence interval) can be easily computed. The mean of X in patients of interest is transformed to the scale with zero defined by the mean of the healthy reference group and the unit defined by the mean of the diseased reference group.

In order to describe the normality or abnormality of a patient group under investigation (SP), the comparison with a healthy reference population Ref0 may be considered to be sufficient. The deviation of SP from normality may be quantified by the standard deviation score as follows:

SDS_X_ = (E[X|SP] − E[X|Ref0])/SD[X|Ref0],

where SD[X|Ref0] is the standard deviation of X in the population Ref0. As for PCI, the zero on the scale of SDS is the mean of X in the population Ref0, but the unit is the standard deviation of X in Ref0, and hence, it is independent of any other reference population. If X is normally distributed, SDS_X_ can be interpreted in terms of percentiles. For example, SDS_X_ = 2.33 would imply that the values of X in SP are centred at the 99^th^ percentile of the values of X in the healthy reference group. In this sense, SDS_X_ < SDS_Y_ allows for researchers to say that the deviation of SP from normality is higher with respect to variable Y than it is with respect to variable X.

The point is, however, that a certain difference from normality does not necessarily imply clinically relevant illness. Patients with SDS_X_ = 3 may feel very sick while those with SDS_Y_ = 4 in the same population (and with a normal X) are quite comfortable, and only those with an SDS_Y_ of 5 or higher may begin to feel that something might be wrong. As well, equal SDS values for different variables are not necessarily associated with comparable prognoses, e.g., in terms of hazard ratios for mortality risk. For this reason, PCI contains more clinically relevant information. For example, if E[X|Ref1] > E[X|Ref0] for the diseased reference population Ref1 and X measures or is associated with, e.g., reduced quality of life or an increased mortality risk, then PCI_X_ = 100 allows for the inference that the patients of interest are as ill as the (well-studied) diseased reference group with respect to variable X. Put differently, this means that their deviation from normality with respect to variable X is clinically meaningful.

**Figure 4:**
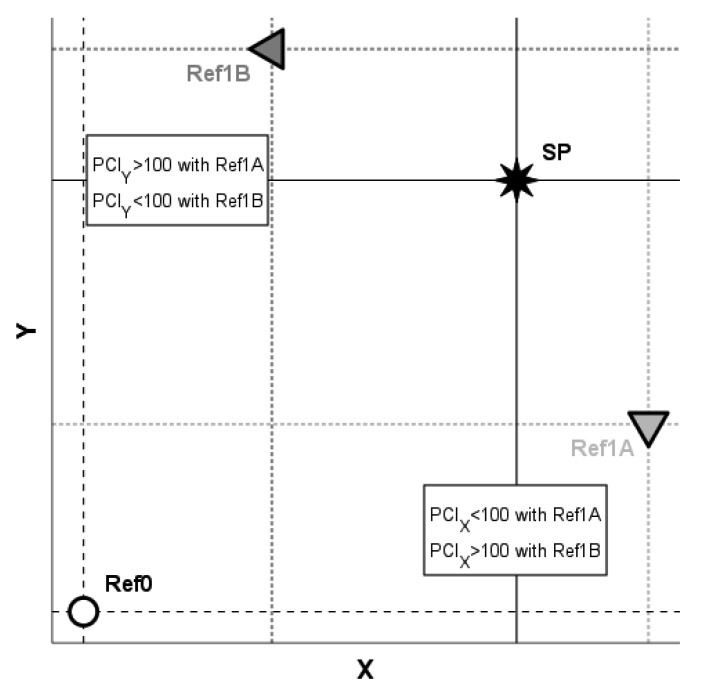
Schematic illustration of the dependence of the comparison of the PCIs of different variables on the choice of the diseased reference population. If Ref1A is chosen as the diseased reference population, then PCI_X_ < PCI_Y_. If Ref1B is chosen as the diseased reference population, then PCI_X_ > PCI_Y_.

We, therefore, recommend considering the presentation of PCI with respect to a carefully chosen diseased reference population instead of using only SDS or other measures solely describing differences from normal reference values. In order to purposefully make use of PCI, we suggest paying attention to the following points:

(i) The sizes of the reference samples Ref0’ and Ref1’ should be sufficient to ensure the representativeness for populations Ref0 and Ref1, respectively.
(ii) The variables X should be considered for the computation of PCI_X_ only if the difference E[X|Ref1] − E[X|Ref0] is clinically meaningful. Of note, if the reference samples are large enough, the estimate of this difference may become significant event if its size is clinically irrelevant. In such a situation, one may obtain estimates for a PCI_X_^ of, e.g., 200, 500, 1000, or even higher, which may sound unpleasant, while the estimate for E[X|SP] − E[X|Ref0] is still of modest clinical importance. This type of misleading presentation should be avoided.
(iii) If the values of the variables being considered depend upon well-known covariables (the most common are certainly sex and age), an appropriately stratified version of PCI should be used.
(iv) When the PCIs of different variables are compared to each other, the results of such comparisons depend upon the choice of the diseased reference population Ref1 and may be materially altered when another population is instead used. For a schematic illustration, please refer to the example in Figure 4. With the diseased reference group Ref1A, we obtain E[X|Ref0] < E[X|SP] < E[X|Ref1A], which yields PCI_X_ < 100, and we obtain E[Y|Ref0] < E[Y|Ref1A] < E[Y|SP], which yields PCI_Y_ > 100, and hence, PCI_X_ < PCI_Y_. In contrast, with the alternative choice of Ref1B as the diseased reference population, E[X|Ref0] < E[X|Ref1B] < E[X|SP] implies PCI_X_ > 100 and E[Y|Ref0] < E[Y|SP] < E[Y|Ref1B] leads to PCI_Y_ < 100, and consequently, PCI_X_ > PCI_Y_. Hence, to avoid arriving at ambiguity, the choice of the diseased reference population should be well-motivated; for example, the same or similar pathomechanisms occurring in both SP and Ref1 may provide a sound reasoning.

It is important to emphasize that PCI was designed for application in the context of clinical and population epidemiology settings. Hence, it should be applied to populations but not to individual patients. For example, if X is a measure of physical functioning and PCIX^ = 60 in a large sample SP’, we can then expect that the average cost per patient associated with physical limitations in the population SP is approximately 60 percent of that cost in population Ref1. On the other hand, if we assume that SP’ consists of only one patient (nSP’ = 1), we can then formally compute the estimate of PCI. However, a PCIX^ = 60 would not mean that the cost associated with the physical disabilities of this individual patient would be 60 percent of the average cost in Ref1 because there is uncertainty in a single value of X in a particular patient. The value of X may intra-individually strongly vary over time, and there is some variability in the measurement conditions if X is obtained from functional testing. There are also subjective components if X is obtained from a questionnaire. Hence, PCIX^ is quite a good estimate of the population mean when computed as an average from a large sample SP’ of a patient population, but it is an unreliable estimate for characterizing an individual patient. We, therefore, strongly discourage the use of PCI for the clinical assessment of individual patients.

## 6. Conclusions

Here we proposed a new measure to compare a population of interest with reference populations. The population comparison index (PCI_X_) comprises thre components: a quantitative variable X that captures the extent of a certain illness; a healthy reference population defining the zero of the scale of PCI; and a well-studied diseased reference population defining the unit of the PCI scale. The value of PCI_X_ (Unit: %) reports to what extent the patients of interest are suffering compared to the diseased reference group as measured by X: not at all (0%), partially (between 0 and 100%), to a similar extent (100%), or to an even larger extent (>100%).

Another well-established index, the standard deviation score (SDS_X_), uses a single reference population (i.e., usually healthy subjects or the general population), where the zero is the mean and the unit is the standard deviation of X in the reference sample. SDS_X_ reports, how far a subject or a group of subjects is from the centre of the reference population, but it does not inform us, whether this deviation is associated with a significant clinical condition. In contrast, PCI_X_ does provide such information, because the value 100% represents the average burden of disease in the patient reference population, and hence, PCI_X_ says how many percent of this burden are present in the patient group of interest.

The stratified version of PCI allows for adjustment for one or more covariables that are potentially relevant for the values of X and for the burden of disease.

The computation of PCI, including its confidence interval, is easily carried out using common statistical software.

In summary, we suggest routinely using PCI, solely or in addition to established measures like SDS, to characterize the “deviation from normality” in epidemiological research.

## Data Availability

No data are necessary for the theoretical considerations in this article. Example 1 solely uses summary data that was published in the cited references. Example 2 uses data of the STAAB study [9,10] and associated investigations. The summary data necessary for the computation of the point estimates of PCI are available as supplementary material to this article. Case-wise data from the STAAB study are not provided in the context of this publication. They may be made available in the context of collaborations, and requests should be addressed to the STAAB consortium [10].

## Supplementary Materials

Supporting information (numerical data of the issues presented in Figure 2 and Figure 3) can be downloaded at: https://www.mdpi.com/article/10.3390/ijerph20032168/s1.
