# Supplementary material for: The Population Comparison Index: An Intuitive Measure to Calibrate the Extent of Impairments in Patient Cohorts in Relation to Healthy and Diseased Populations
Source: Int J Environ Res Public Health. 2023 Jan 25;20(3):2168. doi: 10.3390/ijerph20032168 (PMC9915257; doi:10.3390/ijerph20032168)
Supplement: Supplementary file 1 [file ijerph-20-02168-s001.zip › Gelbrich_Suppl_1_Contents.pdf]

Description of the supplement material

The supplement material contains data underlying example 2 of the paper.

The first data file contains a table with mean values of the three considered outcome variables in the three cohorts (healthy references, diseased references, and patients under investigation) in the total cohorts and in the strata by sex and age. These mean values are being used for the computation of the point estimates of PCI. For the stratified version of PCI, the numbers of cases in the strata are being used for weighing. The computation is explicitly displayed. The point estimates provided in this file coincide with those displayed in Figure 3 of the main paper.

The further data files in EXCEL format show the distributions of the 1,000 bootstrap estimates of PCI for the three outcome variables considered. The 95 percent confidence limits are highlighted in each of the files. These confidence limits coincide with those displayed in Figure 3 of the main paper.
