# Supplementary material for: The Population Comparison Index: An Intuitive Measure to Calibrate the Extent of Impairments in Patient Cohorts in Relation to Healthy and Diseased Populations
Source: Int J Environ Res Public Health. 2023 Jan 25;20(3):2168. doi: 10.3390/ijerph20032168 (PMC9915257; doi:10.3390/ijerph20032168)
Supplement: Supplementary file 1 [file ijerph-20-02168-s001.zip › Gelbrich_Suppl_2_Means.pdf]

Supplement Table: Data used in example 2. Mean values and number of subjects with valid data for HbA1c, left-ventricular posterior wall thickness (LVPW), and logarithmic E/e' in the healthy and diseased reference cohort and in the patient cohort being studied

		Healthy reference cohort		Diseased reference cohort		Patient cohort being studied	
**Variable**	**Subject**	**Mean**	**N**	**Mean**	**N**	**Mean**	**N**
HbA1c [%]	All	5.42	3,763	6.01	887	5.74	56
	Men ≤55 years	5.34	901	5.81	151	5.33	7
	Men >55 years	5.53	821	6.11	337	6.00	5
	Women ≤55 years	5.28	1,143	5.72	108	5.45	23
	Women >55 years	5.58	898	6.11	291	6.13	21
LVPW [mm]	All	7.55	3,782	8.42	851	8.20	54
	Men ≤55 years	7.88	900	8.50	147	8.39	7
	Men >55 years	8.28	827	8.93	323	8.60	5
	Women ≤55 years	6.87	1,155	7.71	106	7.57	21
	Women >55 years	7.41	900	8.07	275	8.67	21
LN (E/e')	All	1.960	3,719	2.124	810	2.213	54
	Men ≤55 years	1.820	885	1.988	137	2.052	7
	Men >55 years	2.040	802	2.145	311	2.380	5
	Women ≤55 years	1.878	1,137	1.963	98	2.111	21
	Women >55 years	2.130	895	2.230	264	2.329	21

Computation of point estimates of PCI

HbA1c

- not stratified: $(5.74–5.42)/(6.01–5.42) \times 100[\%] = 54\%$
- stratified: $\big((5.33–5.34)/(5.81–5.34)\times 7+(6.00–5.53)/(6.11–5.53)\times 5$
  $+(5.45–5.28)/(5.72–5.28)\times 23+(6.13–5.58)/(6.11–5.58)\times 21\big) / 56 \times 100[\%] = 62\%$

LVPW

- not stratified: $(8.20–7.55)/(8.42–7.55) \times 100[\%] = 75\%$
- stratified: $\big((8.39–7.88)/(8.50–7.88)\times 7+(8.60–8.28)/(8.93–8.28)\times 5$
  $+(7.57–6.87)/(7.71–6.87)\times 21+(8.67–7.41)/(8.07–7.41)\times 21\big) / 54 \times 100[\%] = 122\%$

E/e'

- not stratified: $(2.213–1.960)/(2.124–1.960) \times 100[\%] = 154\%$
- stratified: $\big((2.052–1.820)/(1.988–1.820)\times 7+(2.380–2.040)/(2.145–2.040)\times 5$
  $+(2.111–1.878)/(1.963–1.878)\times 21+(2.329–2.130)/(2.230–2.130)\times 21\big)$
  $/ 54 \times 100[\%] = 232\%$
